# *catena*-Poly[[(2-methyl­benzoato-κ^2^ *O*,*O*′)sodium]-di-μ-aqua-κ^4^ *O*:*O*′]

**DOI:** 10.1107/S1600536810010536

**Published:** 2010-03-27

**Authors:** Muhammad Danish, Iram Saleem, Nazir Ahmad, Abdul Rauf Raza, Wojciech Starosta, Janusz Leciejewicz

**Affiliations:** a Department of Chemistry, University of Sargodha, Sargodha 40100, Pakistan; b Institute of Nuclear Chemistry and Technology, ul. Dorodna 16, 03-195 Warszawa, Poland

## Abstract

In the title coordination polymer, [Na(C_8_H_7_O_2_)(H_2_O)_2_]_*n*_, the cation is chelated by the carboxyl­ate O atoms of the anion in a bidentate mode and is surrounded by the O atoms of four water mol­ecules. The coordination of the Na^+^ cation is distorted octa­hedral. The water mol­ecules bridge adjacent metal cations, forming polymeric layers parallel to (100). The structure is stabilized by an extensive network of O—H⋯O hydrogen bonds.

## Related literature

Tin complexes with organic ligands have been studied intensively due to their biological activity, see, for example: Shahzadi *et al.* (2007 ▶). For 2-methyl­benzoic and 4-methyl­benzoic acids as potent allergic sensitizers when applied to human skin, see: Emmet & Suskind (1973 ▶), and as inhibitors of lettuce fruit germination, see: Reynolds (1978 ▶). Sodium 2-methyl­benzoate has been studied as a precursor in the synthesis of biologically active tin(IV) complexes. For the structure of a sodium complex with a 2-methyl-3,5-dinitro­benzoate ligand, see: Danish *et al.* (2010 ▶).
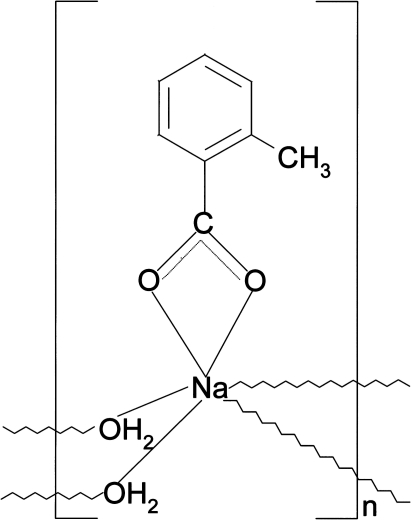


## Experimental

### 

#### Crystal data

[Na(C_8_H_7_O_2_)(H_2_O)_2_]
*M* *_r_* = 194.16
Monoclinic, 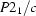

*a* = 16.145 (3) Å
*b* = 8.1155 (16) Å
*c* = 7.3986 (15) Å
β = 92.98 (3)°
*V* = 968.1 (3) Å^3^
*Z* = 4
Mo *K*α radiation
μ = 0.14 mm^−1^
*T* = 293 K
0.55 × 0.41 × 0.11 mm

#### Data collection

Kuma KM-4 four-circle diffractometer
Absorption correction: analytical (*CrysAlis RED*; Oxford Diffraction, 2008 ▶) *T*_min_ = 0.952, *T*_max_ = 0.991
3057 measured reflections
2845 independent reflections
1919 reflections with *I* > 2σ(*I*)
*R*_int_ = 0.024
3 standard reflections every 200 reflections  intensity decay: 0.8%

#### Refinement

*R*[*F* ^2^ > 2σ(*F* ^2^)] = 0.043
*wR*(*F* ^2^) = 0.141
*S* = 1.02
2845 reflections
151 parameters
H atoms treated by a mixture of independent and constrained refinement
Δρ_max_ = 0.44 e Å^−3^
Δρ_min_ = −0.21 e Å^−3^

### 

Data collection: *KM-4 Software* (Kuma, 1996 ▶); cell refinement: *KM-4 Software*; data reduction: *DATAPROC* (Kuma, 2001 ▶); program(s) used to solve structure: *SHELXS97* (Sheldrick, 2008 ▶); program(s) used to refine structure: *SHELXL97* (Sheldrick, 2008 ▶); molecular graphics: *SHELXTL* (Sheldrick, 2008 ▶); software used to prepare material for publication: *SHELXTL*.

## Supplementary Material

Crystal structure: contains datablocks I, global. DOI: 10.1107/S1600536810010536/wm2315sup1.cif

Structure factors: contains datablocks I. DOI: 10.1107/S1600536810010536/wm2315Isup2.hkl

Additional supplementary materials:  crystallographic information; 3D view; checkCIF report

## Figures and Tables

**Table 1:** Selected bond lengths (Å)

Na1—O4	2.3599 (13)
Na1—O4^i^	2.3689 (13)
Na1—O1	2.4141 (13)
Na1—O3	2.4245 (13)
Na1—O3^ii^	2.5086 (13)
Na1—O2	2.5387 (14)

Symmetry codes: (i) 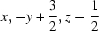; (ii) 
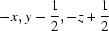
.

**Table 2:** Hydrogen-bond geometry (Å, °)

*D*—H⋯*A*	*D*—H	H⋯*A*	*D*⋯*A*	*D*—H⋯*A*
O4—H42⋯O1^iii^	0.84 (3)	1.94 (3)	2.7582 (17)	163 (2)
O4—H41⋯O2^iv^	0.76 (3)	2.03 (3)	2.7874 (17)	171 (2)
O3—H31⋯O1^v^	0.88 (3)	1.97 (3)	2.7716 (16)	151 (2)
O3—H32⋯O2^i^	0.77 (3)	2.10 (3)	2.8265 (16)	158 (2)

Symmetry codes: (i) 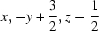; (iii) 
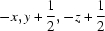
; (iv) 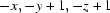; (v) 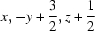.

**Fig. 1.**
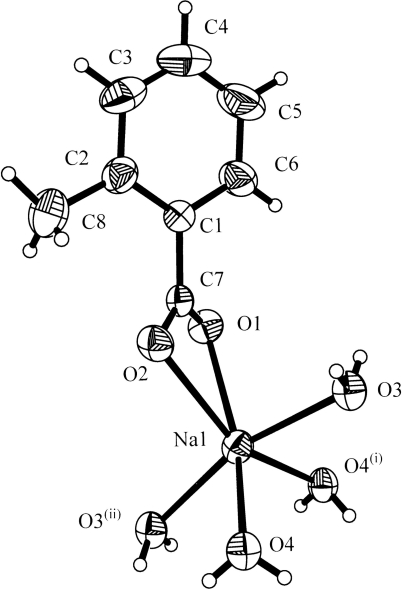
A structural unit of (1) with atom labelling scheme and 50% probability displacement ellipsoids. Symmetry code: (I) x,-y+3/2,z-1;2; (II) -x,y-1/2,-z+1/2.

**Fig. 2.**
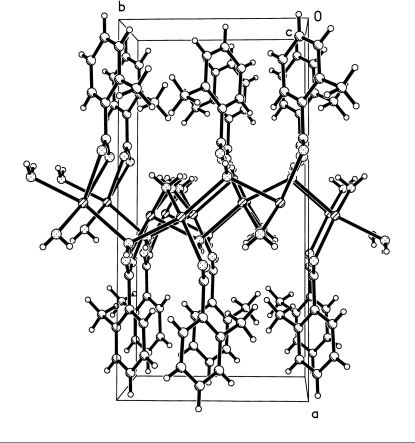
Packing diagram of the structure.

## supplementary crystallographic information

### Comment

2-methylbenzoic and 4-methylbenzoic acids were studied as potent allergic sensitizers when applied to human skin (Emmet & Suskind, 1973). They are also used for the inhibition of lettuce fruit germination (Reynolds, 1978). The title compound was isolated as an intermediate during synthesis of biologically active organotin carboxylates (Shahzadi *et al.*, 2007).

In the polymeric structure of the title compound, [Na(C_8_H_7_O_2_)(H_2_O)_2_]_n_, each sodium ion is coordinated by the carboxylic O atoms of the bidentate anion and by four bridging water O atoms (Fig.1). The coordination geometry around the Na^+^ cation is distorted octahedral with the equatorial plane composed of the carboxylate atoms O1 and O2 and the symmetry-related water O4 and O4^i^ atoms [r.m.s. is 0.0580 (2) Å]. Water O3 and O3^ii^ atoms are at the apical positions. The resulting coordination differs from the one reported in the structure of the Na^+^ complex with the 2-methyl-3,5-dinitrobenzoate anion. Here the metal exhibits coordination number 7 (Danish *et al.*, 2010). The 2-methylbenzoate ring in the title compound is planar with a r.m.s. of 0.0089 (2) Å; the carboxylic group C17/O1/O2 makes an dihedral angle of 37.1 (2)° with the aromatic ring. Na^+^ cations form sheets parallel to the (100) plane in which they are grouped into pairs (Fig. 2). In such a pair, Na^+^ cations are coordinated by ligands with their 2-methylbenzoate rings pointing in the same direction but twisted by an angle of 73.0 (2)° relative to each other. Water O atoms bridge in two directions: *via* O4 atoms along the *b* axis and *via* O3 atoms along the *c* axis. Water molecules act as donors and carboxylate O atoms as acceptors in a network of O—H··· O hydrogen bonds that consolidate the crystal structure. Geometrical parameters of the hydrogen bonding are listed in Table 2.

### Experimental

50 ml of an aqueous solution containing 0.0147 mmol of 2-methylbenzoic acid were added dropwise with continuous stirring at room trmperature to 50 ml of an aqueous solution of sodium bicarbonate (0.0147 mmol). The mixture was then refluxed for 3 hours, cooled to room temperature and concentrated under reduced pressure to afford a dry solid mass which was then purified by re-crystallization from a distilled water-ethanol (4:1) mixture to obtain single crystals.

### Refinement

Water H atoms were localized from Fourier maps and refined isotropically without constraints. H atoms attached to toluene-ring C atoms were positioned geometrically and refined with a riding model.

### Crystal data

**Table:**

[Na(C_8_H_7_O_2_)(H_2_O)_2_]	*F*(000) = 408
*M**_r_* = 194.16	*D*_x_ = 1.332 Mg m^−^^3^
Monoclinic, *P*2_1_/*c*	Mo *K*α radiation, λ = 0.71073 Å
Hall symbol: -P 2ybc	Cell parameters from 25 reflections
*a* = 16.145 (3) Å	θ = 6–15°
*b* = 8.1155 (16) Å	µ = 0.14 mm^−^^1^
*c* = 7.3986 (15) Å	*T* = 293 K
β = 92.98 (3)°	Block, colourless
*V* = 968.1 (3) Å^3^	0.55 × 0.41 × 0.11 mm
*Z* = 4	

### Data collection

**Table:**

Kuma KM-4 four-circle diffractometer	1919 reflections with *I* > 2σ(*I*)
Radiation source: fine-focus sealed tube	*R*_int_ = 0.024
graphite	θ_max_ = 30.1°, θ_min_ = 1.3°
profile data from ω/2θ scans	*h* = −22→22
Absorption correction: analytical (*CrysAlis RED*; Oxford Diffraction, 2008)	*k* = 0→11
*T*_min_ = 0.952, *T*_max_ = 0.991	*l* = −10→0
3057 measured reflections	3 standard reflections every 200 reflections
2845 independent reflections	intensity decay: 0.8%

### Refinement

**Table:**

Refinement on *F*^2^	Primary atom site location: structure-invariant direct methods
Least-squares matrix: full	Secondary atom site location: difference Fourier map
*R*[*F*^2^ > 2σ(*F*^2^)] = 0.043	Hydrogen site location: inferred from neighbouring sites
*wR*(*F*^2^) = 0.141	H atoms treated by a mixture of independent and constrained refinement
*S* = 1.02	*w* = 1/[σ^2^(*F*_o_^2^) + (0.0889*P*)^2^ + 0.1369*P*] where *P* = (*F*_o_^2^ + 2*F*_c_^2^)/3
2845 reflections	(Δ/σ)_max_ < 0.001
151 parameters	Δρ_max_ = 0.44 e Å^−^^3^
0 restraints	Δρ_min_ = −0.21 e Å^−^^3^

### Special details

**Table:**

Geometry. All esds (except the esd in the dihedral angle between two l.s. planes) are estimated using the full covariance matrix. The cell esds are taken into account individually in the estimation of esds in distances, angles and torsion angles; correlations between esds in cell parameters are only used when they are defined by crystal symmetry. An approximate (isotropic) treatment of cell esds is used for estimating esds involving l.s. planes.
Refinement. Refinement of *F*^2^ against ALL reflections. The weighted *R*-factor wR and goodness of fit *S* are based on *F*^2^, conventional *R*-factors *R* are based on *F*, with *F* set to zero for negative *F*^2^. The threshold expression of *F*^2^ > σ(*F*^2^) is used only for calculating *R*-factors(gt) etc. and is not relevant to the choice of reflections for refinement. *R*-factors based on *F*^2^ are statistically about twice as large as those based on *F*, and *R*- factors based on ALL data will be even larger.

### Fractional atomic coordinates and isotropic or equivalent isotropic displacement parameters (Å^2^)

**Table:**

	*x*	*y*	*z*	*U*_iso_*/*U*_eq_	
Na1	0.01791 (3)	0.64831 (7)	0.25611 (7)	0.03320 (17)	
O2	0.14852 (7)	0.53347 (14)	0.41532 (13)	0.0386 (2)	
C7	0.18004 (8)	0.53264 (15)	0.26404 (16)	0.0273 (2)	
C1	0.27247 (8)	0.53757 (16)	0.25234 (17)	0.0305 (3)	
C2	0.32656 (10)	0.4527 (2)	0.3718 (2)	0.0425 (3)	
C6	0.30354 (11)	0.6271 (3)	0.1107 (2)	0.0492 (4)	
C3	0.41092 (11)	0.4583 (3)	0.3414 (3)	0.0562 (5)	
C8	0.29779 (16)	0.3518 (4)	0.5275 (4)	0.0852 (9)	
H8A	0.2774	0.4239	0.6181	0.128*	
H8B	0.3434	0.2884	0.5788	0.128*	
H8C	0.2542	0.2789	0.4848	0.128*	
C5	0.38805 (13)	0.6352 (3)	0.0885 (4)	0.0694 (6)	
C4	0.44096 (12)	0.5494 (3)	0.2038 (4)	0.0672 (6)	
O1	0.13648 (6)	0.53062 (13)	0.11780 (13)	0.0367 (2)	
O3	0.08470 (7)	0.91596 (13)	0.25956 (15)	0.0365 (2)	
O4	−0.05771 (7)	0.74541 (15)	0.49901 (14)	0.0357 (2)	
H32	0.1128 (15)	0.918 (3)	0.179 (4)	0.055 (6)*	
H31	0.1175 (16)	0.921 (3)	0.357 (4)	0.069 (7)*	
H41	−0.0863 (15)	0.676 (3)	0.526 (3)	0.057 (7)*	
H42	−0.0901 (14)	0.819 (3)	0.458 (3)	0.056 (6)*	
H5	0.2640 (13)	0.693 (3)	0.026 (3)	0.056 (6)*	
H2	0.4464 (15)	0.395 (3)	0.412 (3)	0.060 (6)*	
H4	0.4058 (17)	0.705 (4)	−0.018 (4)	0.092 (9)*	
H3	0.495 (2)	0.556 (4)	0.179 (4)	0.096 (9)*	

### Atomic displacement parameters (Å^2^)

**Table:**

	*U*^11^	*U*^22^	*U*^33^	*U*^12^	*U*^13^	*U*^23^
Na1	0.0366 (3)	0.0334 (3)	0.0296 (3)	0.0032 (2)	0.0015 (2)	0.0004 (2)
O2	0.0421 (5)	0.0471 (6)	0.0270 (4)	−0.0009 (4)	0.0063 (4)	0.0023 (4)
C7	0.0323 (6)	0.0244 (5)	0.0251 (5)	−0.0006 (4)	0.0012 (4)	0.0000 (4)
C1	0.0319 (6)	0.0309 (6)	0.0286 (6)	−0.0002 (5)	0.0002 (5)	−0.0024 (5)
C2	0.0395 (7)	0.0431 (8)	0.0439 (8)	0.0023 (6)	−0.0073 (6)	0.0027 (6)
C6	0.0441 (8)	0.0588 (10)	0.0452 (9)	−0.0034 (7)	0.0089 (7)	0.0115 (7)
C3	0.0379 (8)	0.0628 (12)	0.0664 (12)	0.0075 (7)	−0.0114 (8)	−0.0096 (9)
C8	0.0650 (13)	0.106 (2)	0.0834 (16)	0.0098 (13)	−0.0112 (12)	0.0597 (15)
C5	0.0497 (11)	0.0876 (16)	0.0730 (14)	−0.0130 (10)	0.0234 (10)	0.0141 (12)
C4	0.0341 (8)	0.0854 (16)	0.0830 (15)	−0.0045 (9)	0.0118 (9)	−0.0171 (12)
O1	0.0374 (5)	0.0440 (6)	0.0279 (5)	0.0005 (4)	−0.0045 (4)	−0.0024 (4)
O3	0.0474 (6)	0.0369 (5)	0.0253 (5)	−0.0008 (4)	0.0018 (4)	0.0001 (4)
O4	0.0415 (5)	0.0336 (5)	0.0320 (5)	−0.0002 (5)	0.0009 (4)	−0.0016 (4)

### Geometric parameters (Å, °)

**Table:**

Na1—O4	2.3599 (13)	C2—C8	1.506 (3)
Na1—O4^i^	2.3689 (13)	C6—C5	1.384 (3)
Na1—O1	2.4141 (13)	C6—H5	1.02 (2)
Na1—O3	2.4245 (13)	C3—C4	1.367 (4)
Na1—O3^ii^	2.5086 (13)	C3—H2	0.91 (3)
Na1—O2	2.5387 (14)	C8—H8A	0.9600
Na1—C7	2.7787 (14)	C8—H8B	0.9600
Na1—Na1^i^	4.0508 (8)	C8—H8C	0.9600
Na1—Na1^iii^	4.0508 (8)	C5—C4	1.366 (4)
Na1—Na1^ii^	4.0990 (8)	C5—H4	1.02 (3)
Na1—Na1^iv^	4.0991 (8)	C4—H3	0.90 (3)
O2—C7	1.2534 (16)	O3—Na1^iv^	2.5087 (13)
C7—O1	1.2596 (16)	O3—H32	0.77 (3)
C7—C1	1.4999 (18)	O3—H31	0.88 (3)
C1—C6	1.390 (2)	O4—Na1^iii^	2.3689 (13)
C1—C2	1.392 (2)	O4—H41	0.76 (3)
C2—C3	1.393 (3)	O4—H42	0.84 (3)
			
O4—Na1—O4^i^	102.97 (4)	O2—Na1—Na1^iv^	119.11 (3)
O4—Na1—O1	155.22 (5)	C7—Na1—Na1^iv^	117.81 (3)
O4^i^—Na1—O1	100.99 (4)	Na1^i^—Na1—Na1^iv^	65.37 (2)
O4—Na1—O3	86.57 (5)	Na1^iii^—Na1—Na1^iv^	67.05 (2)
O4^i^—Na1—O3	83.84 (5)	Na1^ii^—Na1—Na1^iv^	163.72 (3)
O1—Na1—O3	89.82 (4)	C7—O2—Na1	87.34 (8)
O4—Na1—O3^ii^	85.39 (5)	O2—C7—O1	122.18 (12)
O4^i^—Na1—O3^ii^	85.70 (5)	O2—C7—C1	120.18 (11)
O1—Na1—O3^ii^	102.67 (4)	O1—C7—C1	117.62 (12)
O3—Na1—O3^ii^	165.05 (4)	O2—C7—Na1	65.88 (8)
O4—Na1—O2	102.67 (5)	O1—C7—Na1	60.19 (7)
O4^i^—Na1—O2	152.54 (4)	C1—C7—Na1	158.26 (9)
O1—Na1—O2	52.67 (4)	C6—C1—C2	119.88 (15)
O3—Na1—O2	88.04 (4)	C6—C1—C7	117.16 (13)
O3^ii^—Na1—O2	106.03 (4)	C2—C1—C7	122.92 (13)
O4—Na1—C7	128.38 (5)	C1—C2—C3	117.90 (16)
O4^i^—Na1—C7	125.85 (5)	C1—C2—C8	123.11 (16)
O1—Na1—C7	26.92 (4)	C3—C2—C8	118.97 (17)
O3—Na1—C7	83.36 (4)	C5—C6—C1	120.70 (18)
O3^ii^—Na1—C7	111.48 (4)	C5—C6—H5	119.4 (12)
O2—Na1—C7	26.78 (3)	C1—C6—H5	119.8 (12)
O4—Na1—Na1^i^	125.73 (4)	C4—C3—C2	121.71 (18)
O4^i^—Na1—Na1^i^	30.99 (3)	C4—C3—H2	119.7 (15)
O1—Na1—Na1^i^	74.72 (3)	C2—C3—H2	118.5 (15)
O3—Na1—Na1^i^	67.88 (3)	C2—C8—H8A	109.5
O3^ii^—Na1—Na1^i^	107.18 (3)	C2—C8—H8B	109.5
O2—Na1—Na1^i^	122.27 (3)	H8A—C8—H8B	109.5
C7—Na1—Na1^i^	96.45 (4)	C2—C8—H8C	109.5
O4—Na1—Na1^iii^	31.13 (3)	H8A—C8—H8C	109.5
O4^i^—Na1—Na1^iii^	124.09 (4)	H8B—C8—H8C	109.5
O1—Na1—Na1^iii^	125.87 (3)	C4—C5—C6	119.3 (2)
O3—Na1—Na1^iii^	69.30 (4)	C4—C5—H4	124.9 (16)
O3^ii^—Na1—Na1^iii^	108.49 (3)	C6—C5—H4	115.8 (16)
O2—Na1—Na1^iii^	76.42 (3)	C5—C4—C3	120.39 (17)
C7—Na1—Na1^iii^	99.38 (4)	C5—C4—H3	115 (2)
Na1^i^—Na1—Na1^iii^	131.91 (3)	C3—C4—H3	125 (2)
O4—Na1—Na1^ii^	105.63 (4)	C7—O1—Na1	92.89 (8)
O4^i^—Na1—Na1^ii^	106.05 (4)	Na1—O3—Na1^iv^	112.37 (5)
O1—Na1—Na1^ii^	73.32 (3)	Na1—O3—H32	107.0 (18)
O3—Na1—Na1^ii^	161.66 (4)	Na1^iv^—O3—H32	111.3 (18)
O3^ii^—Na1—Na1^ii^	33.16 (3)	Na1—O3—H31	107.4 (17)
O2—Na1—Na1^ii^	76.09 (3)	Na1^iv^—O3—H31	111.9 (17)
C7—Na1—Na1^ii^	78.33 (3)	H32—O3—H31	106 (2)
Na1^i^—Na1—Na1^ii^	112.95 (2)	Na1—O4—Na1^iii^	117.88 (5)
Na1^iii^—Na1—Na1^ii^	114.63 (2)	Na1—O4—H41	107.1 (18)
O4—Na1—Na1^iv^	66.94 (3)	Na1^iii^—O4—H41	110.8 (17)
O4^i^—Na1—Na1^iv^	63.62 (3)	Na1—O4—H42	107.3 (15)
O1—Na1—Na1^iv^	119.76 (4)	Na1^iii^—O4—H42	108.5 (16)
O3—Na1—Na1^iv^	34.47 (3)	H41—O4—H42	104 (2)
O3^ii^—Na1—Na1^iv^	130.62 (4)		

Symmetry codes: (i) *x*, −*y*+3/2, *z*−1/2; (ii) −*x*, *y*−1/2, −*z*+1/2; (iii) *x*, −*y*+3/2, *z*+1/2; (iv) −*x*, *y*+1/2, −*z*+1/2.

### Hydrogen-bond geometry (Å, °)

**Table:**

*D*—H···*A*	*D*—H	H···*A*	*D*···*A*	*D*—H···*A*
O4—H42···O1^iv^	0.84 (3)	1.94 (3)	2.7582 (17)	163 (2)
O4—H41···O2^v^	0.76 (3)	2.03 (3)	2.7874 (17)	171 (2)
O3—H31···O1^iii^	0.88 (3)	1.97 (3)	2.7716 (16)	151 (2)
O3—H32···O2^i^	0.77 (3)	2.10 (3)	2.8265 (16)	158 (2)

Symmetry codes: (iv) −*x*, *y*+1/2, −*z*+1/2; (v) −*x*, −*y*+1, −*z*+1; (iii) *x*, −*y*+3/2, *z*+1/2; (i) *x*, −*y*+3/2, *z*−1/2.
